# Modeling and Performance Study of Vehicle-to-Infrastructure Visible Light Communication System for Mountain Roads

**DOI:** 10.3390/s24175541

**Published:** 2024-08-27

**Authors:** Wei Yang, Haoran Liu, Guangpeng Cheng

**Affiliations:** Institute of Intelligent Communication and Computing, School of Information and Communication Engineering, Beijing Information Science and Technology University, Beijing 102206, China; haoranliuuu@bistu.edu.cn (H.L.);

**Keywords:** average path loss, channel capacity, outage probability, received power, visible light communication (VLC), vehicle-to-infrastructure (V2I)

## Abstract

Visible light communication (VLC) is considered to be a promising technology for realizing intelligent transportation systems (ITSs) and solving traffic safety problems. Due to the complex and changing environment and the influence of weather and other aspects, there are many problems in channel modeling and performance analysis of vehicular VLC. Unlike existing studies, this study proposes a practical vehicle-to-infrastructure (V2I) VLC propagation model for a typical mountain road. The model consists of both line-of-sight (LOS) and non-line-of-sight (NLOS) links. In the proposed model, the effects of vehicle mobility and weather conditions are considered. To analyze the impact of the considered propagation characteristics on the system, closed-form expressions for several performance metrics were derived, including average path loss, received power, channel capacity, and outage probability. Furthermore, to verify the accuracy of the derived theoretical expressions, simulation results were presented and analyzed in detail. The results indicate that, considering the LOS link and when the vehicle is 50 m away from the infrastructure, the difference in channel gain between moderate fog and dense fog versus clear weather conditions is 1.8 dB and 3 dB, respectively. In addition, the maximum difference in total received optical power between dense fog conditions and clear weather conditions can reach 76.2%. Moreover, under clear weather conditions, the channel capacity when vehicles are 40 m away from infrastructure is about 98.9% lower than when they are 10 m away. Additionally, the outage probability shows a high correlation with the threshold data transmission rate. Therefore, the considered propagation characteristics have a significant impact on the performance of V2I–VLC.

## 1. Introduction

Traffic accidents are a major problem that endangers personal safety and are also a development challenge in countries around the world. In all age groups, road traffic injuries are the 12th leading cause of death [1]. Nearly 1.35 million people die or become disabled in traffic accidents each year, with about 3700 deaths per day in fatal accidents alone [2]. As the main cause of death and disability, road traffic injuries have also caused huge economic losses to society. To address this issue, many researchers are currently focusing on how to improve the transportation system. Among them, vehicle-to-infrastructure (V2I), vehicle-to-vehicle (V2V), and infrastructure-to-vehicle (I2V) communication based on wireless communication technology are considered effective means for lane keeping, collision warning, assisted braking, dynamic direction stability assistance, and traction control. However, the additional hardware facilities will lead to a significant increase in the cost and energy consumption of vehicles and infrastructures. Therefore, visible light communication (VLC), with numerous advantages, has attracted widespread attention in vehicle communication.

VLC, as one of the emerging candidate technologies for wireless access to intelligent transportation systems (ITSs) [3], uses light-emitting diodes (LEDs) to transmit information and illuminate. Visible light is a part of the spectrum of electromagnetic waves with a wavelength range of 380–750 nm and a frequency range of 430–790 THz. As a result, with its huge available bandwidth, VLC is currently able to achieve data transfer rates of up to tens of Gb/s [4]. According to the usage scenario, VLC can mainly be used for communication in indoor and outdoor scenes. Indoor VLC has gained more popularity and expansion due to the success of Light Fidelity (Li-Fi) [5]. However, outdoor VLC has grown slowly owing to challenging environments and mobility and weather constraints [6].

In the context of vehicle communication, V2V–VLC or V2I–VLC systems mostly use LEDs as transmitters (Txs), including LED-based vehicle headlights and taillights, traffic signals, or street lights. Photodetectors (PDs) or complementary metal-oxide semiconductor (CMOS) camera image sensors are used as the receivers (Rxs). In this paper, the focus is on the modeling and performance of V2I–VLC, considering both line-of-sight (LOS) and non-line-of-sight (NLOS) links for mountain roads. Realistic outdoor propagation characteristics are also considered, which is an important area for traffic safety. To illustrate the contribution of the work in this paper, the related works will be first discussed.

### 1.1. Related Works

There are many research directions in outdoor VLC. In [7,8,9], Vieira et al. verified the feasibility of VLC in vehicular networks and ITS and also did some hardware experiments and prototype system simulations. In [10], Vieira et al. improved their previous work and presented a cooperative vehicular traffic scenario. Similarly, Cailean et al. also investigated cooperative communication between vehicles and road infrastructure and demonstrated that the proposed device is suitable for transmitting data using VLC technology [11]. In [12], Liu et al. investigated a hybrid communication system based on VLC and radio frequency (RF). The system can provide location-based services as well as improve the broadcasting downlink efficiency of the traffic broadcast system. In [13,14], Irfan et al. studied the effect of realistic optical interference on vehicular VLC and proposed different models for analyzing the performance of VLC systems. In [15,16], Sharda et al. investigated reflecting intelligent surfaces (RISs)-assisted vehicular VLC. The application of optical RIS can overcome the practical limitation of LOS blockage in V2V–VLC and enhance the reliability of transmission. In [17,18], Yahia et al. introduced the application and development of V2V–VLC, utilizing Multiple-Input Multiple-Output (MIMO). In addition, they analyzed the effect of lateral movement of the vehicle and refracted light on the system. In [19], Aly et al. studied the effect of using different lens combinations on vehicular VLC systems in outdoor environments. In addition, there are many other research directions in vehicular VLC, such as system architecture [20,21,22], signal modulation and demodulation methods [23,24,25], multi-user networks [26,27], MAC layer protocols [28], angle diversity reception [29], etc.

Among the numerous research directions of VLC, one of the important research issues is to develop realistic outdoor VLC channel models for analyzing various performance metrics of vehicular VLC networks. To this end, some efforts have been made in [30,31,32,33,34,35,36,37,38,39,40]. From the perspective of VLC scenarios, in [30], Eldeeb et al. performed channel modeling and performance analysis of I2V–VLC, and also focused on the performance of I2V–VLC systems with access points in the form of streetlights, and derived an approximate closed-form bit error rate (BER) expression. In [31], to study ITS suitable for road safety applications, Kumar et al. used the Lambertian radiation model to model the VLC channel and implemented hardware system simulation using two development kits in field programmable gate array (FPGA). In [32], unlike the general studies using PDs as Rx, Yamazato et al. employed an image sensor-based Rx for vehicle VLC and simultaneously performed optical flow measurements for vehicles in three VLC scenarios: I2V, V2I, and V2V. Moreover, most of the existing studies are based on V2V–VLC scenarios. In [33,34], Viriyasitavat et al. derived a system model for V2V–VLC and conducted a performance evaluation study using empirical data obtained from real-world or measured headlight beam models. The results show that the existing optical channel models cannot accurately estimate the system channel behavior. In [35,36,37], Kim et al. focused on the effect of fog and rain on V2V–VLC performance. In [35], experimental demonstrations on V2V–VLC under fog conditions were conducted and a solution to effectively alleviate the effects of fog by using Fresnel lenses and multiple PDs was proposed. However, the authors only simulated and analyzed the hardware system without giving the channel modeling. In [36], Elamassie et al. quantified the performance of the V2V–VLC link using the ray tracing optical design software Zemax^®^. The proposed link used car high beams as Txs under clear weather, rain, and fog conditions. However, the final expression for path loss is obtained through data fitting, so the expression cannot reflect the functional relationship between the parameters of the proposed system and path loss. Similarly, in [37], a study by Eldeeb et al. also used Zemax^®^’s non-sequential ray tracing tool for channel modeling, and Mie scattering was considered to simulate the weather conditions of clear weather, rain, and fog. Furthermore, the study derived the path loss exponent as a function of distance and different weather conditions. In [38], Mishra et al. used an NS3.25 simulator for realistic channel modeling of VLC-based V2V connection, simulated the system for two modulation methods, open-keyed and variable pulse code modulation, and investigated the proposed system. Most of the above studies consider only LOS links, while, in [39], a model of a V2V–VLC system that considers the effect of atmospheric turbulence (AT) and shadowing was proposed. Under this model, Sharda et al. considered both LOS and NLOS links and proposed a novel Rx architecture using the diversity-multiplexing tradeoff (DMT). In [40], Xie et al. took the Internet of Vehicles as the starting point and used the Lambertian radiation model for channel modeling to analyze the reliability performance of ultrareliable and low-latency communications (URLLC) in outdoor V2V–VLC systems. A comparison of the proposed V2I–VLC model in this paper with the existing research results is shown in Table 1.

### 1.2. Contributions

Based on the literature review above, it can be found that most of the existing studies are based on some idealized assumptions or ignore the impact of natural conditions on outdoor VLC systems and so their conclusions cannot fully reflect all the characteristics of vehicle-mounted VLC systems in specific scenarios. In addition, most studies focus on describing channel characteristics rather than quantifying the factors that affect the VLC channel model. The main contributions in this paper can be listed as follows:

1. A typical V2I–VLC communication scenario on a mountain road is considered. This consideration is more practical and specific, thus reflecting the V2I–VLC performance in real application scenarios.
2. In the proposed scenario, the outdoor VLC channel is modeled taking into account AT and multiple propagation characteristics. In addition, vehicle mobility is considered in the modeling process, and dynamic V2I–VLC models are emphasized.
3. Based on the channel model, closed-form expressions for the performance metrics of the LOS and NLOS links in the proposed scenario are derived. These performance metrics include average path loss, received optical power, channel capacity, and outage probability.
4. Further, the derived theoretical expressions are compared with numerical simulation results in order to verify the accuracy of the derived theoretical expressions.
5. Furthermore, the proposed propagation model is thoroughly investigated and analyzed in this paper, as well as compared with existing studies.

By modeling the channel of the proposed V2V–VLC system and deriving closed-form expressions for performance indicators, it can be concluded that the considered propagation characteristics have a significant impact on the average path loss, received optical power, channel capacity, and outage probability of the V2I–VLC system. The research content and conclusions will provide strong support for subsequent work and the deployment of the infrastructure for the V2I–VLC system.

### 1.3. Paper Organization

The rest of this paper is organized as follows. In Section 2, the system model and channel model of V2I–VLC are discussed. In Section 3, the performance analysis methods are discussed and closed-form expressions for the performance metrics, such as average path loss, received optical power, channel capacity, and outage probability, are derived. In Section 4, numerical results are presented. In Section 5, some discussions are provided. Finally, summary and conclusions are given in Section 6.

## 2. System Model and Channel Modeling Methods

**Figure 1:**
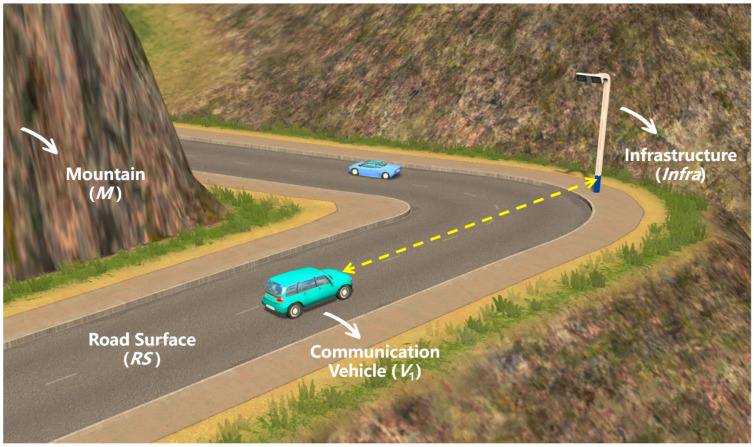
Schematic of a typical mountain road V2I–VLC communication scenario.

In this section, the proposed V2I–VLC scenario, system model, and channel model are introduced and discussed in detail. As shown in Figure 1, a typical V2I–VLC communication scenario on a mountain road is considered, where the infrastructure *Infra* is located on the roadside at the turn of the road. The resulting V2I–VLC scenarios will include both LOS and NLOS situations. The V2I–VLC system models based on LOS and NLOS will be discussed later, respectively. As shown in Figure 1, the communication vehicle *V*_1_ is located on the road *RS* and is traveling in the direction of *Infra*. The two headlights of *V*_1_ are used as Txs, namely Tx1 and Tx2, and the PD-based Rx is deployed on the *Infra* at the same height as Tx1 and Tx2 (relative to *RS*). Mountain *M* is located on the left side of *V*_1_.

The data to be sent are modulated on both Tx1 and Tx2 by the on-off keying (OOK) method. Two headlights emit light with modulation information simultaneously, forming LOS and NLOS links. The LOS link light is directed to the PD-based Rx surface, while the NLOS link light is reflected by the *RS* or *M* and then irradiated on the Rx surface. As a result, the Rx can receive LOS and NLOS rays, as well as noise.

### 2.1. System Model

#### 2.1.1. System Model for LOS Scenarios

**Figure 2:**
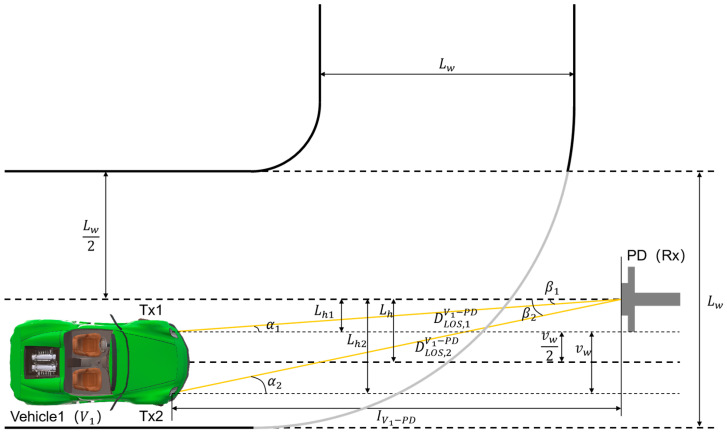
Schematic of V2I–VLC system model for LOS scenario.

Take the case that *V*_1_ is located on the right side of the Rx axis as an example. The V2I–VLC system model in the LOS scenario is shown in Figure 2. The definitions of left or right are based on the driver’s viewpoint. The vehicle width of *V*_1_ is $v_{w}$. The road width is $L_{w}$. The PD-based Rx is outside the road and facing the center of the road where *V*_1_ is located. The emergent angles at Tx1 and Tx2 are denoted as $\alpha_{1}$ and $\alpha_{2}$, respectively. The incident angles at Rx are denoted by $\beta_{1}$ and $\beta_{2}$. Moreover, considering that Txs and Rx have the same height concerning *RS*, it follows that $\alpha_{1}=\beta_{1}$ and $\alpha_{2}=\beta_{2}$. In addition, due to the characteristics of VLC, the light irradiated to Rx must satisfy a certain condition in order to receive the LOS signal, i.e., $\cos\alpha_{j}\geq \cos\Psi_{PD}$, where $\Psi_{PD}$ is the field-of-view (FOV) of Rx. The longitudinal distance of light propagation between *V*_1_ and Rx is $I_{V_{1}-PD}$, and the transverse distance is $L_{h}$ (using the direction of vehicle travel as the longitudinal direction, and vehicle movement from side to side as the transverse direction). Additionally, the lateral displacement of the vehicle corresponding to the $j^{th}$ Tx is $L_{h_{j}}$. Therefore, the LOS propagation distance for the $j^{th}$ Tx can be expressed as $D_{LOS,j}^{V_{1}-PD}=\sqrt{{L_{h_{j}}}^{\;2}+{I_{V_{1}-PD}}^{\;2}}$. Furthermore, in the proposed model, to emphasize the realistic traffic environment, the movement of vehicles is considered to be random. Consequently, the range of values of $L_{h}$ can be expressed as $0\leq L_{h}\leq \left(L_{w}/2-v_{w}/2\right)$.

#### 2.1.2. System Model for NLOS Scenarios

**Figure 3:**
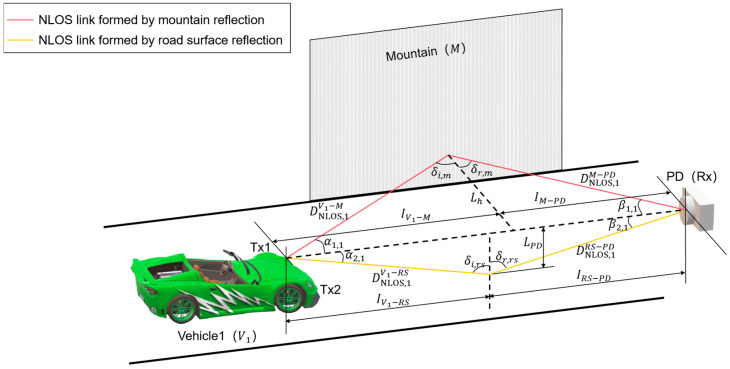
Schematic of V2I–VLC system model for NLOS scenario.

The V2I–VLC system model for the NLOS scenario is shown in Figure 3. Taking the rays emitted from Tx1 as an example, two NLOS links are mainly considered for the proposed V2I–VLC scenario, namely, the NLOS link formed by the reflection of *M* (as shown by the red line in Figure 3), and the NLOS link formed by the reflection of *RS* (as shown by the yellow line in Figure 3).

**Figure 4:**
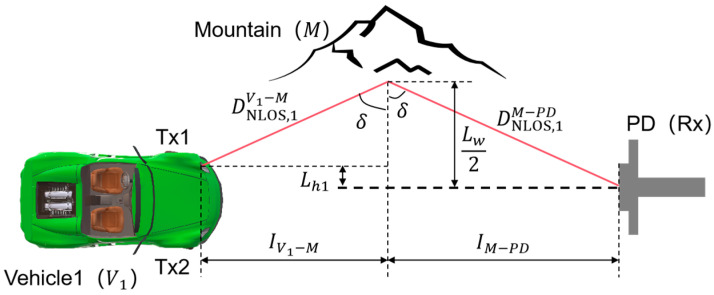
Schematic model of the V2I–VLC system in the NLOS scenario formed by *M* reflection.

Now, consider the above two NLOS links separately. The schematic of the NLOS scenario formed by *M* reflection is shown in Figure 4 (taking the example that Tx1 is located to the left of the Rx axis). For the rays from Tx1, the NLOS propagation distances from *V*_1_ to *M* and from *M* to Rx are denoted as $D_{NLOS,1}^{V_{1}-M}$ and $D_{NLOS,1}^{M-PD}$. The rays from the headlights fall on *M* and are reflected. Denote the angles of incidence and reflection as $\delta_{i,m}$ and $\delta_{r,m}$, as shown in Figure 3. By the Law of Reflection, it follows that $\delta_{i,m}=\delta_{r,m}=\delta$. Based on the reflection points, the longitudinal distances of light propagation from *V*_1_ to *M* and from *M* to Rx can be expressed as $I_{V_{1}-M}$ and $I_{M-PD}$. Also, it can be obtained that $I_{V_{1}-PD}=I_{V_{1}-M}+I_{M-PD}$. In addition, the NLOS scenario formed by the rays emitted from Tx2 and reflected by *M* is similar to the situation of Tx1.

**Figure 5:**
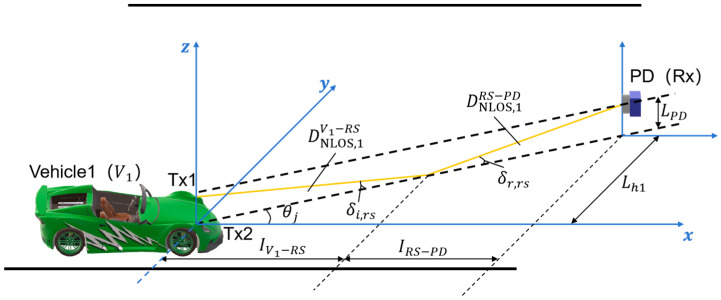
Schematic model of the V2I–VLC system in the NLOS scenario formed by *RS* reflection.

The reflection phenomenon also exists when the headlights’ rays fall on the RS. The schematic of the NLOS scenario formed by the reflection of the *RS* is shown in Figure 5 (taking the example that Tx1 is located to the right of the Rx axis). For the rays from Tx1, the NLOS propagation distances from *V*_1_ to *RS* and from *RS* to Rx are denoted as $D_{NLOS,1}^{V_{1}-RS}$ and $D_{NLOS,1}^{RS-PD}$. The rays from the headlights strike *RS* and are reflected. Denote the angles of incidence and reflection as $\delta_{i,rs}$ and $\delta_{r,rs}$; it follows that $\delta_{i,rs}=\delta_{r,rs}$. Based on the reflection point on RS, the longitudinal distances of light propagation from *V*_1_ to *RS* and from *RS* to Rx are denoted as $I_{V_{1}-RS}$ and $I_{RS-PD}$, respectively, and $I_{V_{1}-PD}=I_{V_{1}-RS}+I_{RS-PD}$. In addition, the height of Rx from the ground is the same as the height of Tx, which is expressed as $L_{PD}$. Similarly, the NLOS link from Tx2 can be studied.

### 2.2. Channel Modeling Methods

Currently, there are two main approaches to model VLC channels for V2I or V2V. One is to apply the Beer–Lambert formula [31,41] based on the Lambert source assumption. In this method, the radiation of Tx is required to obey the Lambertian radiation model, and then the angular distribution of the radiation intensity is modeled using the generalized Lambertian radiation intensity [42]. Then, the channel DC gain (optical power gain) and system impulse response (CIR) expressions are further obtained. The other method of VLC channel modeling pursues more realistic results. Researchers create a simulation environment in software such as OpticStudio^®^ or Zemax^®^. Then, a type of non-sequential ray tracing is applied to the asymmetric light source to obtain data for calculating the CIR. Finally, these data are imported into Matlab^®^ software to construct the CIR [43,44].

However, the above two channel modeling approaches are mainly applied in indoor VLC systems and extended to outdoor VLC studies. The main difference between outdoor and indoor VLC is the propagation characteristics of light. Outdoor VLC is subject to sunlight, rain, fog, and AT [37,45,46]. Therefore, in [47,48], the authors proposed an outdoor vehicular VLC channel modeling under the influence of AT. This channel model considers the effect of AT in the outdoor environment, which is more realistic than the two methods mentioned above and is used in this paper. Considering the short transmission distance of the proposed V2I–VLC, the effects of AT are modeled using a weak turbulence mechanism, i.e., the received signal at Rx is as follows:

(1) $$y=\eta \sum_{j=1}^{2}h_{j}x+n,$$

where η (in A/W) is the photoelectric conversion efficiency of the PD; $h_{j}$ is the real-valued channel coefficient of the link between the $j^{th}$ Tx and Rx, which can be modeled as follows:

(2) $$h_{j}=h_{a}h^{PL\_av},$$

where $h^{PL\_av}$ denotes the average path loss. $h_{a}$ is the channel coefficient due to AT, which is generally modeled as a lognormal distribution with variance ${\sigma_{l}}^{\;2}$, and its probability density function (PDF) is as follows:

(3) $$f_{h_{a}}(h_{a})=\frac{1}{2h_{a}\sqrt{2\pi {\sigma_{y}}^{\;2}}}e^{-\frac{{\left[\ln\left(h_{a}\right)+2{\sigma_{y}}^{\;2}\right]}^{2}}{8{\sigma_{y}}^{\;2}}}\;\mathrm{for}\;h_{a}>0,$$

where ${\sigma_{l}}^{\;2}=4{\sigma_{y}}^{\;2}$. In addition, n in (1) denotes zero-mean additive Gaussian white noise (AWGN) with variance ${\sigma_{o}}^{\;2}$. The shot noise (with variance $\sigma_{\mathrm{shot}}^{2}$) and the thermal noise (with variance $\sigma_{\mathrm{thermal}}^{2}$) caused by sunlight and ambient light are the main noises affecting the quality of outdoor V2I–VLC, and that ${\sigma_{o}}^{\;2}$ is expressed as follows:

(4) $${\sigma_{o}}^{\;2}=\sigma_{\mathrm{shot}}^{2}+\sigma_{\mathrm{thermal}}^{2}.$$

According to [33,49], $\sigma_{\mathrm{shot}}^{2}$ and $\sigma_{\mathrm{thermal}}^{2}$ are shown in (5) and (6):

(5) $$\sigma_{\mathrm{shot}}^{2}=2q\eta P_{r}B+2qI_{bg}I_{2}B,$$

(6) $$\sigma_{\mathrm{thermal}}^{2}=\frac{8\pi kT_{k}}{G}\eta_{c}A_{r}I_{2}B^{2}+\frac{16\pi^{2}kT_{k}\Gamma }{g_{m}}\eta_{c}^{2}A_{r}^{2}I_{3}B^{3},$$

where q is the amount of electronic charge; η is the photoelectric conversion efficiency of PD; $P_{r}$ is the average received optical power; B is the system bandwidth; $I_{bg}$ is the received background noise current; $I_{2}$ is the noise bandwidth factor of background noise; k is the Boltzmann constant; $T_{k}$ is the absolute temperature; G is the open-loop voltage gain; $\eta_{c}$ is the fixed capacitance per unit area of PD; Γ is the field-effect transistor (FET) channel noise factor; $g_{m}$ is the FET transconductance; $I_{3}$ is the FET channel noise current for thermal noise.

## 3. Performance Analysis Methods

### 3.1. Expressions for Path Loss and Average Path Loss

In this section, the V2I–VLC path loss expressions for LOS and NLOS links are derived based on the channel modeling in Section 2, and (5) and (9) in [48]. The weather factor and other propagation characteristics are taken into account. In addition, novel closed-form expressions for the average path loss are derived.

Taking the LOS link as an example, the path loss of the proposed outdoor VLC consists of two main components, which are geometric loss and attenuation loss. The geometric loss is caused by the diffusion of emitted rays to areas larger than the PD receiving aperture. The geometric loss between the $j^{th}$ Tx and PD can be expressed as follows:

(7) $$PL_{geo,j}=10\log_{10}\left\{\frac{D_{R}\left[\cos{\left(\alpha_{j}\right)}^{1/\varepsilon }\right]}{\zeta D_{LOS,j}^{V_{1}-PD}}\right\}\;\mathrm{for}\;j=1,2,$$

where $D_{R}$ denotes the aperture diameter of the PD; ε and ζ are weather-related correction factors [43] (pp. 6892–6894). The attenuation loss is the result of scattering and absorption. It is assumed that most of the scattered photons cannot be captured by the PD and that some of the scattered rays may be received by the PD after reflection. Based on the Beer–Lambert formula and the assumption above, the attenuation loss can be defined as follows:

(8) $$PL_{att,j}=10\log_{10}\left\{\exp\left[-c_{E}D_{LOS,j}^{V_{1}-PD}{\left(\frac{D_{R}}{\zeta D_{LOS,j}^{V_{1}-PD}}\right)}^{\varepsilon /2}\right]\right\}\;\mathrm{for}\;j=1,2,$$

where $c_{E}$ denotes the extinction coefficient for a given weather type; $D_{LOS,j}^{V_{1}-PD}{\left(\frac{D_{R}}{\zeta D_{LOS,j}^{V_{1}-PD}}\right)}^{\varepsilon /2}$ denotes an additional term proportional to the geometric spread of the light source. In summary, considering the dual Txs of vehicle *V*_1_ and the geometric relation $\alpha_{j}=\arccos\frac{I_{V_{1}-PD}}{D_{LOS,j}}$, the overall path loss of the LOS link can be expressed as follows:

(9) $$h_{LOS}^{PL}=\frac{1}{2}\sum_{j=1}^{2}\left[\frac{{D_{R}}^{\;2}{\left(I_{V_{1}-PD}\right)}^{2/\varepsilon }}{\zeta^{2}{\left(D_{LOS,j}^{V_{1}-PD}\right)}^{2+2/\varepsilon }}e^{-c_{E}D_{LOS,j}^{V_{1}-PD}{\left(\frac{D_{R}}{\zeta D_{LOS,j}^{V_{1}-PD}}\right)}^{\varepsilon /2}}\right].$$

In the proposed V2I–VLC system model, the path loss of the NLOS links is divided into two parts, *M* and *RS*, based on the reflected objects. In the same way, the overall path loss of the NLOS links can be expressed as follows:

(10) $$h_{NLOS\_M}^{PL}=\frac{1}{2}\sum_{j=1}^{2}\left\{\left[\frac{{A_{M}}^{\;2}{\left(I_{V_{j}-M}\right)}^{2/\varepsilon }}{\zeta^{2}{\left(D_{NLOS,j}^{V_{1}-M}\right)}^{2+2/\varepsilon }}\right]\left[\frac{{D_{R}}^{\;2}{\left(I_{M-PD}\right)}^{2/\varepsilon }}{\zeta^{2}{\left(D_{NLOS,j}^{M-PD}\right)}^{2+2/\varepsilon }}\right]e^{-c_{E}\left(D_{NLOS,j}^{V_{1}-M}+D_{NLOS,j}^{M-PD}\right){\left[\frac{D_{R}}{\zeta \left(D_{NLOS,j}^{V_{1}-M}+D_{NLOS,j}^{M-PD}\right)}\right]}^{\varepsilon /2}}\right\},$$

and

(11) $$h_{NLOS\_RS}^{PL}=\frac{1}{2}\sum_{j=1}^{2}\left\{\left[\frac{{A_{RS}}^{\;2}{\left(I_{V_{j}-RS}\right)}^{2/\varepsilon }}{\zeta^{2}{\left(D_{NLOS,j}^{V_{1}-RS}\right)}^{2+2/\varepsilon }}\right]\left[\frac{{D_{R}}^{\;2}{\left(I_{RS-PD}\right)}^{2/\varepsilon }}{\zeta^{2}{\left(D_{NLOS,j}^{RS-PD}\right)}^{2+2/\varepsilon }}\right]e^{-c_{E}\left(D_{NLOS,j}^{V_{1}-RS}+D_{NLOS,j}^{RS-PD}\right){\left[\frac{D_{R}}{\zeta \left(D_{NLOS,j}^{V_{1}-RS}+D_{NLOS,j}^{RS-PD}\right)}\right]}^{\varepsilon /2}}\right\},$$

where $A_{M}$ and $A_{RS}$ denote the effective areas (assumed to be circular) of *M* and *RS* illuminated by the rays, respectively.

#### 3.1.1. The Average Path Loss for LOS Links

The rays from the LOS link are the main source of received power. From (9), it can be seen that the average path loss of the LOS link is closely related to $D_{LOS,j}^{V_{1}-PD}$, and $D_{LOS,j}^{V_{1}-PD}=\sqrt{{L_{h_{j}}}^{\;2}+{I_{V_{1}-PD}}^{\;2}}$. $L_{h_{j}}$ takes on different ranges of values when Tx1 is located at different positions relative to the center axis of PD. Therefore, the average path loss for LOS links needs to be discussed separately. Refer to Appendix A for the detailed derivation process. Therefore, the closed-form expression for the average path loss of the LOS link is obtained as follows:

(12) $$\begin{array}{ll} h_{LOS}^{PL\_av} & =\frac{{D_{R}}^{\;2}e^{-\frac{c_{E}D_{R}}{\zeta }}}{2\zeta^{2}I_{V_{1}-PD}\left(L_{w}-v_{w}\right)Gamma\left[1+\frac{1}{\varepsilon }\right]}\{2\sqrt{\pi }Gamma\left[\frac{1}{2}+\frac{1}{\varepsilon }\right]\\ & -Gamma\left[1+\frac{1}{\varepsilon }\right]\left\{Beta\left[\frac{4{I_{V_{1}-PD}}^{\;2}}{4{I_{V_{1}-PD}}^{\;2}+{L_{w}}^{\;2}},\frac{1}{2}+\frac{1}{\varepsilon },\frac{1}{2}\right]+Beta\left[\frac{4{I_{V_{1}-PD}}^{\;2}}{4{I_{V_{1}-PD}}^{\;2}+{\left(L_{w}-2v_{w}\right)}^{2}},\frac{1}{2}+\frac{1}{\varepsilon },\frac{1}{2}\right]\right\}\}. \end{array}$$

#### 3.1.2. The Average Path Loss of the NLOS Link Formed by *M*

In the proposed V2I–VLC system, the NLOS link light caused by *M* and *RS* reflections also has a large impact on the system performance. Using similar analytical methods, the overall average path loss closure expression for the NLOS link formed by *M* can be expressed as follows:

(13) $$\begin{array}{ll} h_{NLOS\_M}^{PL\_av} & =\frac{{A_{M}}^{\;2}{D_{R}}^{\;2}I_{V_{1}-PD}e^{\frac{-c_{E}D_{R}}{\zeta }}}{2\left(L_{w}-v_{w}\right)\zeta^{4}}\{\frac{\left\{Beta\left[\frac{4{I_{V_{1}-PD}}^{\;2}}{{L_{w}}^{\;2}+4{I_{V_{1}-PD}}^{\;2}},\frac{3}{2}+\frac{2}{\varepsilon },\frac{1}{2}\right]-Beta\left[\frac{{I_{V_{1}-PD}}^{\;2}}{{L_{w}}^{\;2}+{I_{V_{1}-PD}}^{\;2}},\frac{3}{2}+\frac{2}{\varepsilon },\frac{1}{2}\right]\right\}}{{\left(I_{V_{1}-M,1,case1}I_{M-PD,1,case1}\right)}^{2}}\\ & +\frac{\left\{Beta\left[\frac{4{I_{V_{1}-PD}}^{\;2}}{{\left(3L_{w}-2v_{w}\right)}^{2}+4{I_{V_{1}-PD}}^{\;2}},\frac{3}{2}+\frac{2}{\varepsilon },\frac{1}{2}\right]-Beta\left[\frac{{I_{V_{1}-PD}}^{\;2}}{{L_{w}}^{\;2}+{I_{V_{1}-PD}}^{\;2}},\frac{3}{2}+\frac{2}{\varepsilon },\frac{1}{2}\right]\right\}}{{\left(I_{V_{1}-M,1,case2}I_{M-PD,1,case2}\right)}^{2}}\\ & +\frac{\left\{Beta\left[\frac{4{I_{V_{1}-PD}}^{\;2}}{{\left(L_{w}+2v_{w}\right)}^{2}+4{I_{V_{1}-PD}}^{\;2}},\frac{3}{2}+\frac{2}{\varepsilon },\frac{1}{2}\right]-Beta\left[\frac{{I_{V_{1}-PD}}^{\;2}}{{L_{w}}^{\;2}+{I_{V_{1}-PD}}^{\;2}},\frac{3}{2}+\frac{2}{\varepsilon },\frac{1}{2}\right]\right\}}{{\left(I_{V_{2}-M,2,case1}I_{M-PD,2,case1}\right)}^{2}}\\ & +\frac{\left\{Beta\left[\frac{{I_{V_{1}-PD}}^{\;2}}{{L_{w}}^{\;2}+{I_{V_{1}-PD}}^{\;2}},\frac{3}{2}+\frac{2}{\varepsilon },\frac{1}{2}\right]-Beta\left[\frac{{I_{V_{1}-PD}}^{\;2}}{4{L_{w}}^{\;2}+{I_{V_{1}-PD}}^{\;2}},\frac{3}{2}+\frac{2}{\varepsilon },\frac{1}{2}\right]\right\}}{{\left(I_{V_{2}-M,2,case2}I_{M-PD,2,case2}\right)}^{2}}\}. \end{array}$$

Note that the derivation process of the above expression is different from the LOS link. The cases of Tx1 and Tx2 need to be considered separately. Refer to Appendix B for the specific derivation procedure.

#### 3.1.3. The Average Path Loss of the NLOS Link Formed by *RS*

Similarly, based on the symmetry of Tx1 and Tx2, from Tx1, the average path loss of the NLOS link formed by RS can be mathematically calculated. Referring to Appendix C, the overall average path loss of the closed-form expression for the NLOS link formed by *RS* can be obtained as follows:

(14) $$\begin{array}{ll} h_{NLOS\_RS}^{PL\_av} & =\frac{8{A_{RS}}^{\;2}{D_{R}}^{\;2}{I_{V_{1}-PD}}^{\;4/\varepsilon }e^{\frac{-c_{E}D_{R}}{\zeta }}}{\zeta^{4}P^{3/2+2/\varepsilon }\left(L_{w}-v_{w}\right)Gamma\left[2+\frac{2}{\varepsilon }\right]}\{2\sqrt{\pi }Gamma\left[\frac{3}{2}+\frac{2}{\varepsilon }\right]\\ & -Gamma\left[2+\frac{2}{\varepsilon }\right]\left\{Beta\left[\frac{4P}{4P+{L_{w}}^{\;2}},\frac{3}{2}+\frac{2}{\varepsilon },\frac{1}{2}\right]+Beta\left[\frac{4P}{4P+{\left(L_{w}-2v_{w}\right)}^{2}},\frac{3}{2}+\frac{2}{\varepsilon },\frac{1}{2}\right]\right\}\}, \end{array}$$

where $P={I_{V_{1}-PD}}^{\;2}+4{L_{PD}}^{\;2}$.

### 3.2. Expressions for Received Optical Power

The received optical power is an important factor in VLC systems. Consider (1), where x denotes the on-off keying (OOK) symbol corresponding to the intensity modulation-direct detection (IM/DD) scheme and y is the received signal at Rx. Accordingly, the received optical power of the rays arriving at the Rx through the LOS/NLOS link can be expressed as follows:

(15) $$P_{r,j}=P_{t,j}h_{j},$$

where $P_{t,j}$ corresponds to the emitted power of the $j^{th}$ headlight LED and $h_{j}$ can be calculated from (2). In the proposed system, $h^{PL\_av}$ denotes the average path loss of the LOS/NLOS link as shown in (12), (13), and (14). Therefore, the power and the total power of the LOS/NLOS link received by Rx from the two Txs can be respectively expressed as follows:

(16) $$P_{r}^{LOS}=\sum_{j=1}^{2}P_{t,j}h_{a}h_{LOS}^{PL\_av}=h_{a}h_{LOS}^{PL\_av}\sum_{j=1}^{2}P_{t,j},$$

(17) $$P_{r}^{NLOS\_M}=\sum_{j=1}^{2}P_{t,j}h_{a}h_{NLOS\_M}^{PL\_av}=h_{a}h_{NLOS\_M}^{PL\_av}\sum_{j=1}^{2}P_{t,j},$$

(18) $$P_{r}^{NLOS\_RS}=\sum_{j=1}^{2}P_{t,j}h_{a}h_{NLOS\_RS}^{PL\_av}=h_{a}h_{NLOS\_RS}^{PL\_av}\sum_{j=1}^{2}P_{t,j},$$

and

(19) $$P_{r}^{Total}=P_{r}^{LOS}+P_{r}^{NLOS\_M}+P_{r}^{NLOS\_RS}.$$

### 3.3. Expressions for Channel Capacity

Channel capacity, as an important metric for performance analysis of communication systems, is the maximum data transmission rate that can be achieved by the communication system. In short-distance and free space optical communication using IM/DD, the average and peak intensity of the transmitted signal is limited [50]. Thus, most of the current research mainly focuses on the bounds of the system capacity, i.e., the emphasis is on deriving the lower/upper bounds [51,52]. In [52], a closed-form expression of channel capacity for VLC systems with high SNR is derived by neglecting the gap between the exact and lower limit values of channel capacity:

(20) $$C=\frac{B}{2\ln2}\ln\left(1+\frac{e\sum_{j=1}^{2}{\mathrm{SNR}}_{j}}{2\pi }\right),$$

where B is the bandwidth; e is the natural constant and ${\mathrm{SNR}}_{j}$ denotes the average received electrical signal-to-noise ratio (SNR) from the $j^{th}$ Tx. For the proposed V2I–VLC system, since the received optical power at Rx can be expressed by (15), ${\mathrm{SNR}}_{j}$ can be expressed as follows:

(21) $${\mathrm{SNR}}_{j}=\frac{{\left(\eta P_{t,j}h_{j}\right)}^{2}}{{\sigma_{o}}^{\;2}}.$$

Substituting (2) and (21) into (20), the channel capacity of the proposed V2I–VLC system can be obtained as follows:

(22) $$C=\frac{B}{2\ln2}\ln\left[1+\frac{e{\left(\eta P_{t,j}h_{a}h^{PL\_av}\right)}^{2}}{\pi {\sigma_{o}}^{\;2}}\right].$$

### 3.4. Expressions for Outage Probability

The outage probability is a key measure of the reliability and stability of a communication system. An outage occurs when the SNR corresponding to all communication links falls below a certain threshold SNR [47].

For the proposed V2I–VLC system, the average received electrical SNR from the $j^{th}$ Tx can be expressed by (21). Define it as follows:

(23) $${\mathrm{SNR}}_{j}=\frac{{\left(\eta P_{t,j}h_{j}\right)}^{2}}{{\sigma_{o}}^{\;2}}≜R_{j}h_{j}^{2}.$$

According to the definition of outage probability, and the threshold data transmission rate $C_{th}$, the threshold SNR can be calculated from (21) and (22) as follows:

(24) $${\mathrm{SNR}}_{th}=\frac{\pi }{e}\left(e^{\frac{2C_{th}\ln2}{B}}-1\right).$$

Consequently, the outage probability of the proposed V2I–VLC system can be expressed as follows:

(25) $$P_{out}=\prod_{j=1}^{2}\int_{0}^{\sqrt{\frac{{\mathrm{SNR}}_{th}}{R_{j}}}}f_{h_{j}}\left(h_{j}\right)dh_{j},$$

where $R_{j}$ is shown in (23). Substituting (2) and (3) into (25) and applying the change of variables method for simplification, the closed-form expression for the outage probability is obtained as follows:

(26) $$P_{out}=\prod_{j=1}^{2}Q\left(-\frac{\ln\frac{\sqrt{{\mathrm{SNR}}_{th}}\sigma_{o}}{h^{PL\_av}\eta P_{t,j}}}{2\sigma_{y}}\right),$$

where Q(⋅) is the Gaussian Q-function that represents the probability that the value of a Gaussian normal random variable is greater than the standard deviation of x, and defined as $Q\left(x\right)=\frac{1}{\sqrt{2\pi }}\int_{x}^{\infty }e^{\frac{-t^{2}}{2}}dt$.

## 4. Results

In this section, the performance of the proposed system in terms of path loss, received power, channel capacity, and outage probability will be demonstrated. To validate the accuracy of the derived theoretical expression in the previous sections, a numerical simulation is conducted using MATLAB^®^ software, version R2023a. Since weather conditions have a significant impact on outdoor V2I–VLC systems, they are considered in the discussion. In the simulation, corresponding to each value of $I_{V_{1}-PD}$, $3\times 10^{6}$ channels are considered. In addition, all the simulation parameters related to the proposed V2I–VLC system are referred to in Table 2.

### 4.1. Average Path Loss

**Figure 6:**
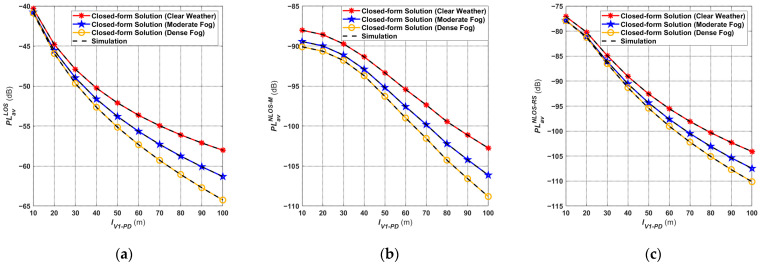
Average path loss of (**a**) LOS link; (**b**) NLOS-M link; (**c**) NLOS-RS link under different weather conditions [(−) sign in the path loss value indicates that the path loss is in the form of a penalty].

In this subsection, the performance of the proposed V2I–VLC model in terms of path loss is presented. Since the proposed V2I–VLC model is based on short-range communication over LEDs, the path loss has a significant impact on the model. Figure 6 illustrates the path loss under different transmission distances $I_{V_{1}-PD}$ in LOS and NLOS links given in (12), (13), and (14) for different weather conditions. The simulation curves are also incorporated to confirm the accuracy of the derived closed-form expression for the average path loss. It is worth noting that the negative sign indicates that the path loss is in the form of a penalty. As a result, the path loss in both the LOS and NLOS links increases with the transmission distance, as shown in Figure 6. From the comparison, it is obvious that the LOS and NLOS links have the highest channel gain in clear weather conditions and the worst gain in dense fog conditions. As an example, for $I_{V_{1}-PD}=50\;m$ in the LOS link, the average path loss value is about −52 dB for clear weather conditions, −53.8 dB for moderate fog conditions, and −55 dB for dense fog conditions. The difference in channel gain between moderate fog and dense fog versus clear weather conditions is 1.8 dB and 3 dB, respectively. In addition, the further *V*_1_ is from Rx (the larger the value of $I_{V_{1}-PD}$), the larger the difference in channel gain described above. Therefore, the transmission distance and the weather factor have significant impacts on the average path loss of the V2I–VLC.

**Figure 7:**
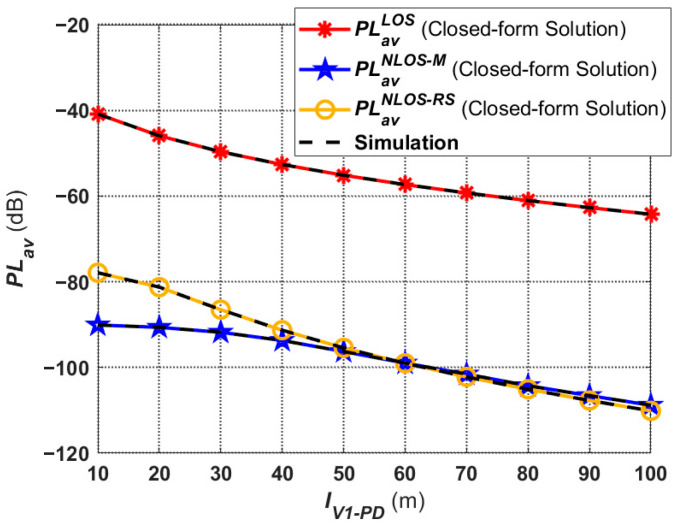
Average path loss corresponding to the proposed V2I–VLC propagation model under fog weather conditions.

The average V2I–VLC path loss in different links under dense fog conditions is shown in Figure 7. It is worth noting that the path loss of NLOS-M links is higher than those of NLOS-RS links when the values of $I_{V_{1}-PD}$ range from 10 to 50 m. However, after 60 m, $PL_{av}^{NLOS\_RS}\approx PL_{av}^{NLOS\_M}$. It shows that, when *V*_1_ is close to Rx, the reflection effect of the road surface plays the leading role compared to the reflection of the mountain, i.e., most of the optical power received by Rx from the NLOS link is reflected by the road surface. Additionally, when *V*_1_ is far away from Rx, the road surface and mountain reflection have basically the same effect on the performance of V2I–VLC.

### 4.2. Received Optical Power

**Figure 8:**
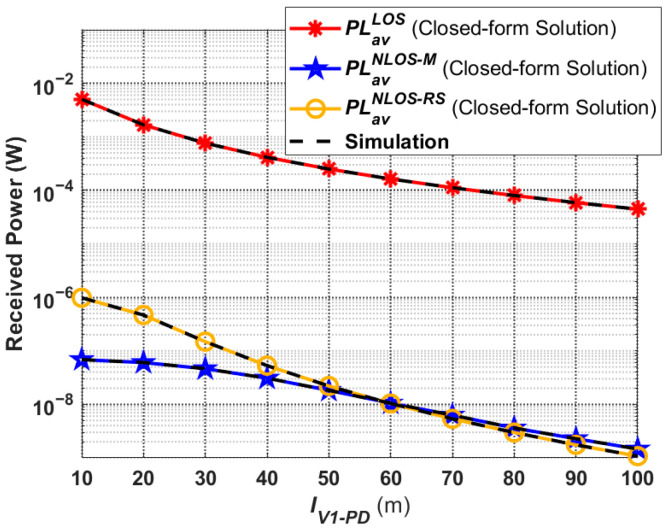
Received optical power corresponding to the proposed V2I–VLC propagation model under moderate fog conditions.

In this subsection, the received optical power performance corresponding to the proposed V2I–VLC propagation model is discussed. Figure 8 gives the received optical power in different links under moderate fog conditions based on (16), (17), and (18). It can be seen that the received optical power is highly correlated with the average path loss, and they are directly proportional to each other. Thus, the trends of the received optical power curves shown in Figure 8 are consistent with those in Figure 7. Meanwhile, in the total received power at Rx, the received optical power from the LOS link is much larger than the received optical power from the two NLOS links. In addition, when *V*_1_ is at a close distance from Rx, under the same conditions, the NLOS power caused by the reflection from *RS* is larger than that caused by the reflection from *M*. This is mainly due to the fact that the NLOS propagation distance of the rays reflected by the road surface ( $D_{NLOS,j}^{V_{1}-RS}$ and $D_{NLOS,j}^{RS-PD}$) is smaller than the NLOS propagation distance of the rays reflected by the mountain ( $D_{NLOS,j}^{V_{1}-M}$ and $D_{NLOS,j}^{M-PD}$) during the light propagation process. Additionally, when *V*_1_ and Rx become gradually farther away from each other, the received optical power of the two NLOS links is also basically the same because the propagation distances of the rays are not very different.

**Figure 9:**
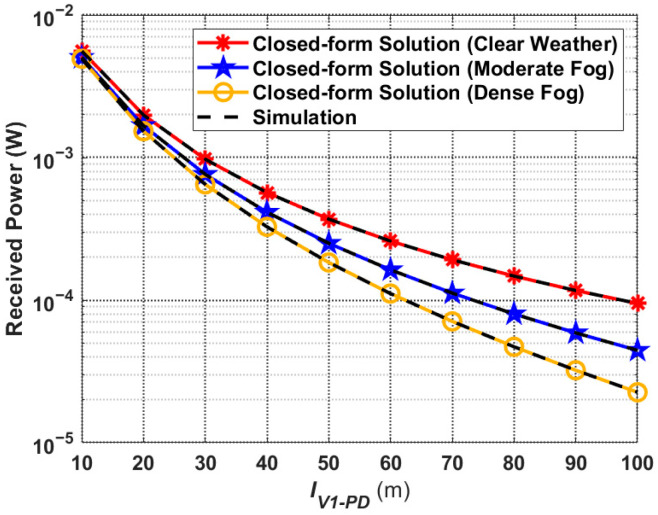
Total received optical power corresponding to the proposed V2I–VLC propagation model under different weather conditions.

The total received optical power of the V2I–VLC system, based on (19) as well as the simulation curves under different weather conditions, are presented in Figure 9. It can be seen that the total received optical power is the largest in clear weather conditions and the smallest in dense fog conditions. Moreover, the further the distance between *V*_1_ and Rx, the larger the difference in total received optical power. For example, when $I_{V_{1}-PD}=10\;m$, the total received optical power in dense fog conditions is only about 12.5% lower than it is in clear weather conditions. However, when $I_{V_{1}-PD}=100\;m$, the ratio reaches 76.2%. This is due to the fact that, the further away *V*_1_ is from Rx and the worse the weather conditions are, the more the light from the headlights is affected, making the performance of the V2I–VLC worse as well.

### 4.3. Channel Capacity

**Figure 10:**
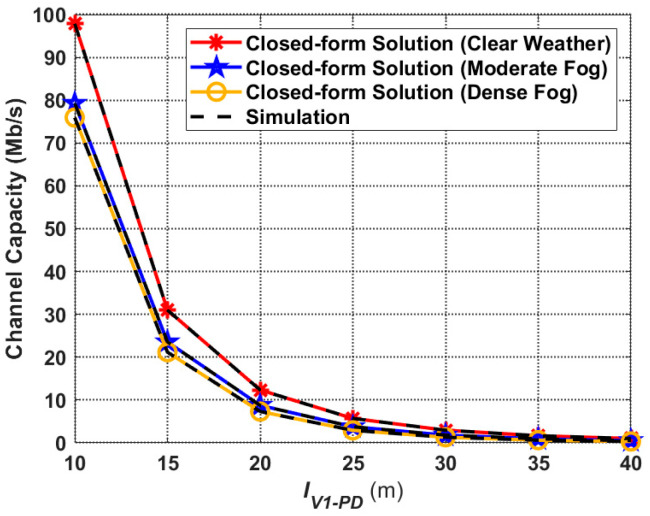
Channel capacity corresponding to the proposed V2I–VLC propagation model for different weather conditions.

In this subsection, the channel capacity performance corresponding to the proposed V2I–VLC propagation model is discussed. Figure 10 illustrates the relationship between the channel capacity (maximum data rate) and the transmission distance based on (22), and also the simulation curves. First, it achieves the highest capacity at all transmission distances in clear weather conditions due to better average path loss performance compared to foggy weather. Second, transmission distance has a great impact on the channel capacity of the proposed V2I–VLC propagation model. For example, under clear weather conditions, the channel capacity at $I_{V_{1}-PD}=20\;m$ is about 87.4% lower than the channel capacity at $I_{V_{1}-PD}=10\;m$. Additionally, when $I_{V_{1}-PD}=40\;m$ (compared to $I_{V_{1}-PD}=10\;m$), this ratio reaches 98.9%. Furthermore, with the increasing transmission distance, the effect of weather conditions on the channel capacity becomes less significant. For instance, Figure 10 shows that when $I_{V_{1}-PD}=10\;m$, the channel capacity of the moderate and dense fog is about 18.6 Mb/s and 21.9 Mb/s lower than that of the clear weather, but, when $I_{V_{1}-PD}=20\;m$, the difference is only 3.6 Mb/s and 5.0 Mb/s. It reveals that, for the proposed V2I–VLC propagation model, the transmission distance is a more influential factor than the weather conditions in terms of the performance of the channel capacity.

### 4.4. Outage Probability

**Figure 11:**
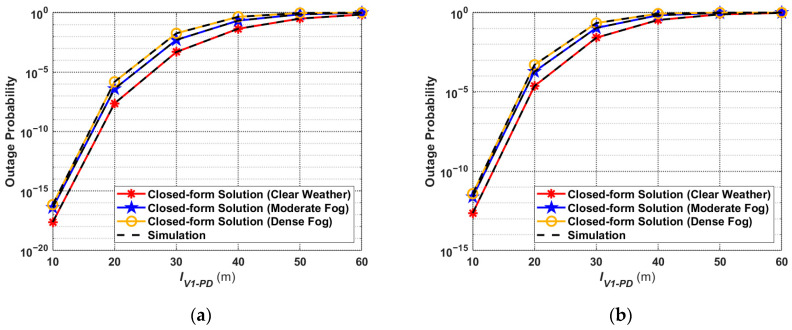
Outage probability for different weather conditions with threshold data transfer rates of (**a**) 200 kb/s and (**b**) 500 kb/s.

Figure 11 shows the relationship between the outage probability and transmission distance, based on (26), and the simulation curves. From (26), it can be seen that the received optical power is highly correlated with the threshold SNR, which is a function of the threshold data transmission rate. Therefore, the threshold of the data transmission rate has a great influence on the outage performance of the V2I–VLC system. Figure 11a,b depict the outage performance when the threshold is 200 kb/s and 500 kb/s, respectively. It is observed that the higher the threshold data transfer rate is set, the higher the outage probability under the same weather and transmission distance conditions. For example, for the same moderate fog conditions and $I_{V_{1}-PD}=30\;m$, the outage probability at a threshold data transfer rate of 200 kb/s is about $5.1\times 10^{-3}$, and 0.11 at 500 kb/s. In addition, it can also be seen from Figure 11 that the outage probability increases rapidly as the transmission distance increases. When considering the weather factor, the outage probability curves for different weather conditions are relatively close to each other, regardless of whether the threshold data transmission rate is 200 kb/s or 500 kb/s. The results indicate that weather conditions have less impact on the outage performance compared to transmission distance.

## 5. Discussion

In order to observe the impact of the considered VLC scheme and the propagation characteristics on the proposed V2I–VLC system, the performance of the system can be compared with similar studies [44,47]. In [44], a general V2I–VLC model is proposed. Additionally, in [47], typical V2V–VLC models based on two-lane roads are considered.

As shown in Figure 4 in [47], under clear weather conditions, the path loss in the LOS scenario is about −44 dB when Tx is 10 m away from Rx. Furthermore, when Tx is 50 m away from Rx, the path loss is −62.5 dB. Comparing the results in Figure 6a and [47], it can be observed that mobility has a large impact on the path loss performance of vehicular VLC systems. This is because, in the proposed V2I–VLC system, the path loss is only about −52.5 dB (under clear weather conditions) when the Tx and Rx are 50 m apart. The biggest difference between the two models is that the Rx of the V2V–VLC system is moving, while the Rx of the V2I–VLC system is fixed.

Comparing the channel capacity shown in Figure 6 in [44] with that of the proposed V2I–VLC system shown in Figure 10 (under clear weather conditions), it can be observed that the use of multiple PDs for signal reception at the Rx end can significantly improve the performance of the vehicular VLC system. This is because Equal Gain Combining (EGC) uses a direct sum of the branch signals from all PDs with equal weighting to all branches, which increases the overall received SNR [44].

In summary, in future research, the focus could be on the real mobility of vehicles or infrastructure. In addition, it will be of great significance to investigate MIMO techniques applicable to vehicular VLC.

## 6. Conclusions

In order to solve the traffic safety problem and improve the transportation system, a V2I–VLC system in typical mountainous road scenarios is studied. In the channel modeling process, the LOS and NLOS links are considered, and propagation characteristics such as AT and other weather factors are included in the system model. In addition, to analyze the performance of the proposed V2I–VLC model, closed-form expressions for the key VLC performance metrics are derived, such as average path loss, received optical power, channel capacity, and outage probability. In order to verify the accuracy of the derived closed-form expressions, Monte Carlo methods are used for stochastic simulation in MATLAB® software, version R2023a. Simulation results show that the closed-form expressions have high accuracy and reference values. Unlike existing studies, the effect of NLOS links on the system model is considered more comprehensively, including the NLOS links formed by reflections from mountains and road surfaces. At the same time, a more realistic computational method is used in the consideration of vehicle mobility. In the study of the relevant performance metrics, it can be found that the considered propagation characteristics have a significant impact on the average path loss and received optical power of V2I–VLC. In the LOS link, when $I_{V_{1}-PD}=50\;m$, the difference in channel gain between moderate fog and dense fog versus clear weather conditions is 1.8 dB and 3 dB, respectively. Additionally, when $I_{V_{1}-PD}=100\;m$, the total received optical power in dense fog conditions is about 76.2% lower than in clear weather conditions. Furthermore, it is found that transmission distance is a more influential factor than weather conditions in terms of channel capacity and outage probability. In clear weather, the channel capacity at $I_{V_{1}-PD}=20\;m$ is about 87.4% lower than that at $I_{V_{1}-PD}=10\;m$. Similarly, the outage probability increases rapidly as transmission distance increases.

However, there are some limitations in our study. For example, the effect of neighboring vehicles is not considered in the proposed model. In addition, the channel capacity is not calculated using an exact closed-form expression. In future studies, new techniques can be proposed to minimize the adverse effects of certain propagation characteristics on V2I–VLC systems. Furthermore, the study of the proposed system and modeling revealed that AT and weather conditions are important factors affecting the performance of V2V–VLC systems. The weather conditions considered in the study are clear weather and fog weather. Therefore, in future studies, the effect of rain or snow weather conditions on the V2V–VLC system can be emphasized. Additionally, other performance metrics applicable to outdoor VLC systems can be further derived.

## Figures and Tables

**Table 1:** Comparison of existing studies with the work performed in this paper.

Classification	[30]	[31]	[32]	[36,37]	[39]	Our Proposed Work
VLC Scenario	I2V	V2I	I2V, V2I and V2V	V2V	V2V	V2I
System Model	Both LOS and NLOS	Only LOS	Only LOS	Only LOS	Both LOS and NLOS	Both LOS and NLOS
Modeling Approach	Using the non- sequential ray tracing function of OpticStudio^®^	Based on Lambertian radiation model and hardware simulation	Based on Image sensors	Using the non- sequential ray tracing function of Zemax^®^	Considering AT and shadow effects	Channel modeling method considering AT
Tx	LED streetlights	LED arrays conforming to Lambertian radiation patterns	LED arrays and LED headlights	LED headlights	LED taillights	LED headlights
Rx	PD-based receivers	PD-based receivers	High-speed camera-based receiver	PD-based receivers	PD-based receivers	PD-based receivers
Comparative Study of LOS and NLOS	×	×	×	×	√	√
Considering AT and Various Propagation Characteristics	×	×	×	√	√	√
Deriving Closed-form Expressions for Channel Models and Performance Indicators	×	√	×	√	×	√

**Table 2:** Modeling and simulation parameters of the proposed V2I–VLC system.

Symbol	Variable Name	Value
$D_{R}$	Aperture Diameter of PD	0.02 m
$L_{w}$	Road Width	4.5 m
$v_{w}$	Vehicle Width	1.8 m
c (Clear Weather)	Extinction Coefficient (Clear Weather)	0
c (Moderate Fog)	Extinction Coefficient (Moderate Fog)	0.00782
c (Dense Fog)	Extinction Coefficient (Dense Fog)	0.01565
ε (Clear Weather)	Weather Correction Factor 1 (Clear Weather)	0.0175
ε (Moderate Fog)	Weather Correction Factor 1 (Moderate Fog)	0.0172
ε (Dense Fog)	Weather Correction Factor 1 (Dense Fog)	0.0170
ζ (Clear Weather)	Weather Correction Factor 2 (Clear Weather)	0.1585
ζ (Moderate Fog)	Weather Correction Factor 2 (Moderate Fog)	0.1600
ζ (Dense Fog)	Weather Correction Factor 2 (Dense Fog)	0.1550
$L_{PD}$	Height of Headlights and PD	0.7 m
${\sigma_{o}}^{\;2}$	Variance of the Zero Mean AWGN Noise	1
${\sigma_{l}}^{\;2}$	Variance of the Lognormal Distribution	0.2
η	Photoelectric Conversion Efficiency of PD	1
$P_{t,j}$	Output Power of a Single Tx	30 W
B	System Bandwidth	20 MHz

## Data Availability

Data are contained within the article.

## Appendix A

1. Case 1 of Tx1: Tx1 is located to the left of the center axis of the PD

As shown in Figure 2, the lateral displacement $L_{h_{1}}$ of the vehicle cannot change instantaneously and substantially due to the limitations of $L_{w}$, $v_{w}$ and the vehicle speed. Therefore, the uniform distribution can be used to model $L_{h_{1}}$, i.e., $L_{h_{1}}∼u\left(0,\frac{L_{w}}{2}\right)$. In addition, since $D_{LOS,1}^{V_{1}-PD}=\sqrt{{L_{h_{1}}}^{\;2}+{I_{V_{1}-PD}}^{\;2}}$, the distribution of $D_{LOS,1}^{V_{1}-PD}$ can be obtained according to the variable transformation method:

(A1)
$$D_{LOS,1,case1}^{V_{1}-PD}=X=\sqrt{{L_{h_{1}}}^{\;2}+{I_{V_{1}-PD}}^{\;2}}∼f_{X}(x)=\frac{2x}{L_{w}\sqrt{x^{2}-{I_{V_{1}-PD}}^{\;2}}},$$

where $x_{1}=I_{V_{1}-PD}\leq x\leq x_{2}=\sqrt{{I_{V_{1}-PD}}^{\;2}+\frac{{L_{w}}^{\;2}}{4}}$.

2. Case 2 of Tx1: Tx1 is located to the right of the center axis of the PD

In this case, since $L_{h_{1}}∼u\left(0,\frac{L_{w}}{2}-v_{w}\right)$, the following can also be obtained:

(A2)
$$D_{LOS,1,case2}^{V_{1}-PD}=Y=\sqrt{{L_{h_{1}}}^{\;2}+{I_{V_{1}-PD}}^{\;2}}∼f_{Y}(y)=\frac{2y}{\left(L_{w}-2v_{w}\right)\sqrt{y^{2}-{I_{V_{1}-PD}}^{\;2}}}$$

where $y_{1}=I_{V_{1}-PD}\leq y\leq y_{2}=\sqrt{{I_{V_{1}-PD}}^{\;2}+{\left(\frac{L_{w}}{2}-v_{w}\right)}^{2}}$.

According to (9), (A1) and (A2), the average path loss of the LOS link from Tx1 is as follows:

(A3)
$$h_{LOS,1}^{PL\_av}=\sum_{case}\left\{\int \left[\frac{{D_{R}}^{\;2}{\left(I_{V_{1}-PD}\right)}^{2/\varepsilon }p_{case}}{\zeta^{2}{\left(D_{LOS,1,case}^{V_{1}-PD}\right)}^{2+2/\varepsilon }}e^{-c_{E}D_{LOS,1}^{V_{1}-PD}{\left(\frac{D_{R}}{\zeta D_{LOS,1,case}^{V_{1}-PD}}\right)}^{\varepsilon /2}}\right]f_{D_{LOS,1}^{V_{1}-PD}}(D_{LOS,1,case}^{V_{1}-PD})dD_{LOS,1,case}^{V_{1}-PD}\right\},$$

where case∈{case1,case2} and $p_{case}$ denote the probability that Tx1 lies to the left or to the right of the center axis of PD. Converting the summation notation in (A3) to the form of the sum of two terms and integrating them separately, the following can be obtained:

(A4)
$$\begin{array}{ll} I & =\frac{{D_{R}}^{\;2}{\left(I_{V_{1}-PD}\right)}^{2/\varepsilon }}{\zeta^{2}\left(L_{w}-v_{w}\right)}\left[\int_{x_{1}}^{x_{2}}\frac{1}{x^{1+2/\varepsilon }\sqrt{x^{2}-{I_{V_{1}-PD}}^{\;2}}}e^{-c_{E}x{\left(\frac{D_{R}}{\zeta x}\right)}^{\varepsilon /2}}dx+\int_{y_{1}}^{y_{2}}\frac{1}{y^{1+2/\varepsilon }\sqrt{x^{2}-{I_{V_{1}-PD}}^{\;2}}}e^{-c_{E}y{\left(\frac{D_{R}}{\zeta y}\right)}^{\varepsilon /2}}dy\right]\\ & ≜\frac{{D_{R}}^{\;2}{\left(I_{V_{1}-PD}\right)}^{2/\varepsilon }}{\zeta^{2}\left(L_{w}-v_{w}\right)}\left(I_{1}+I_{2}\right). \end{array}$$

Since the value of ε is small enough, assuming ${\left(\frac{D_{R}}{\zeta x}\right)}^{\varepsilon /2}\approx \frac{D_{R}}{\zeta x}$ and ${\left(\frac{D_{R}}{\zeta y}\right)}^{\varepsilon /2}\approx \frac{D_{R}}{\zeta y}$, it follows that the following is true:

(A5)
$$\begin{array}{ll} I_{1} & \approx e^{-\frac{c_{E}D_{R}}{\zeta }}\int_{x_{1}}^{x_{2}}\frac{1}{x^{1+2/\varepsilon }\sqrt{x^{2}-{I_{V_{1}-PD}}^{\;2}}}dx\\ & =e^{-\frac{c_{E}D_{R}}{\zeta }}\cdot \frac{{I_{V_{1}-PD}}^{\;-\left(1+2/\varepsilon \right)}\left(\sqrt{\pi }Gamma\left[\frac{1}{2}+\frac{1}{\varepsilon }\right]-Beta\left[\frac{4{I_{V_{1}-PD}}^{\;2}}{4{I_{V_{1}-PD}}^{\;2}+{L_{w}}^{\;2}},\frac{1}{2}+\frac{1}{\varepsilon },\frac{1}{2}\right]Gamma\left[1+\frac{1}{\varepsilon }\right]\right)}{2Gamma\left[1+\frac{1}{\varepsilon }\right]}, \end{array}$$

and

(A6)
$$\begin{array}{ll} I_{2} & \approx e^{-\frac{c_{E}D_{R}}{\zeta }}\int_{y_{1}}^{y_{2}}\frac{1}{y^{1+2/\varepsilon }\sqrt{x^{2}-{I_{V_{1}-PD}}^{\;2}}}dx\\ & =e^{-\frac{c_{E}D_{R}}{\zeta }}\cdot \frac{{I_{V_{1}-PD}}^{\;-\left(1+2/\varepsilon \right)}\left(\sqrt{\pi }Gamma\left[\frac{1}{2}+\frac{1}{\varepsilon }\right]-Beta\left[\frac{4{I_{V_{1}-PD}}^{\;2}}{4{I_{V_{1}-PD}}^{\;2}+{\left(L_{w}-2v_{w}\right)}^{2}},\frac{1}{2}+\frac{1}{\varepsilon },\frac{1}{2}\right]Gamma\left[1+\frac{1}{\varepsilon }\right]\right)}{2Gamma\left[1+\frac{1}{\varepsilon }\right]}. \end{array}$$

In (A5) and (A6), the gamma function Gamma[z] satisfies $\Gamma \left(z\right)=\int_{0}^{\infty }t^{z-1}e^{-t}dt$, and the incomplete beta function Beta[z,a,b] satisfies $B_{z}\left(a,b\right)=\int_{0}^{z}t^{a-1}{\left(1-t\right)}^{b-1}dt$. Substituting (A5) and (A6) into (A4), the average path loss of the LOS link corresponding to Tx1 can be obtained. Based on the symmetry of Tx1 and Tx2 and (A4), the closed-form expression for the average path loss of the LOS link is obtained as follows:

(A7)
$$\begin{array}{ll} h_{LOS}^{PL\_av} & =\frac{{D_{R}}^{\;2}e^{-\frac{c_{E}D_{R}}{\zeta }}}{2\zeta^{2}I_{V_{1}-PD}\left(L_{w}-v_{w}\right)Gamma\left[1+\frac{1}{\varepsilon }\right]}\{2\sqrt{\pi }Gamma\left[\frac{1}{2}+\frac{1}{\varepsilon }\right]\\ & -Gamma\left[1+\frac{1}{\varepsilon }\right]\left\{Beta\left[\frac{4{I_{V_{1}-PD}}^{\;2}}{4{I_{V_{1}-PD}}^{\;2}+{L_{w}}^{\;2}},\frac{1}{2}+\frac{1}{\varepsilon },\frac{1}{2}\right]+Beta\left[\frac{4{I_{V_{1}-PD}}^{\;2}}{4{I_{V_{1}-PD}}^{\;2}+{\left(L_{w}-2v_{w}\right)}^{2}},\frac{1}{2}+\frac{1}{\varepsilon },\frac{1}{2}\right]\right\}\}. \end{array}$$

## Appendix B

1. Case 1 of Tx1: Tx1 is located to the left of the center axis of the PD

The geometrical relationship in Figure 4 shows that $I_{V_{1}-M,1,case1}=\frac{L_{w}-2L_{h_{1}}}{2\left(L_{w}-L_{h_{1}}\right)}I_{V_{1}-PD}$. Therefore, the angle of incidence and reflection $\delta =\arctan\frac{I_{V_{1}-M,1,case1}}{L_{w}/2-L_{h_{1}}}$ can be expressed as $\delta =\arctan\frac{I_{V_{1}-PD}}{L_{w}-L_{h_{1}}}$. It is known that $L_{h_{1}}∼u\left(0,\frac{L_{w}}{2}\right)$, so the distribution of δ can be obtained according to the variable transformation method:

(A8)
$$\delta ∼f_{\Delta }\left(\delta \right)=\frac{4I_{V_{1}-PD}}{L_{w}\left(1-\cos2\delta \right)},$$

where $\arctan\frac{I_{V_{1}-PD}}{L_{w}}\leq \delta <\arctan\frac{2I_{V_{1}-PD}}{L_{w}}$. $D_{NLOS,1,case1}^{V_{1}-M}$ and $D_{NLOS,1,case1}^{M-PD}$ can also be denoted by δ, i.e., $D_{NLOS,1,case1}^{V_{1}-M}=\frac{I_{V_{1}-M,1,case1}}{\sin\delta }$ and $D_{NLOS,1,case1}^{M-PD}=\frac{I_{M-PD,1,case1}}{\sin\delta }$.

2. Case 2 of Tx1: Tx1 is located to the right of the center axis of the PD

Similarly, the geometric relation $I_{V_{1}-M,1,case2}=\frac{L_{w}+2L_{h_{1}}}{2\left(L_{w}+L_{h_{1}}\right)}I_{V_{1}-PD}$ is obtained in this case. At this point, $\delta =\arctan\frac{I_{V_{1}-PD}}{L_{w}+L_{h_{1}}}$. For the sake of distinction, when Tx1 is located to the right of the center axis of PD, δ is denoted as θ, i.e., $\theta =\arctan\frac{I_{V_{1}-PD}}{L_{w}+L_{h_{1}}}$. From $L_{h_{1}}∼u\left(0,\frac{L_{w}}{2}-v_{w}\right)$, the distribution of θ can be obtained according to the variable transformation method:

(A9)
$$\theta ∼f_{\Theta }\left(\theta \right)=\frac{2I_{V_{1}-PD}}{\left(L_{w}-2v_{w}\right)\sin^{2}\theta },$$

where $\arctan\frac{2I_{V_{1}-PD}}{3L_{w}-2v_{w}}\leq \theta \leq \arctan\frac{I_{V_{1}-PD}}{L_{w}}$. In addition, it can also be obtained that $D_{NLOS,1,case2}^{V_{1}-M}=\frac{I_{V_{1}-M,1,case2}}{\sin\theta }$ and $D_{NLOS,1,case2}^{M-PD}=\frac{I_{M-PD,1,case2}}{\sin\theta }$.

According to (10), (A8), and (A9), the average path loss of the NLOS link formed by *M* from Tx1, consists of two components, respectively:

(A10)
$$\begin{array}{ll} I_{1} & =p_{case1}\int_{\delta_{1}}^{\delta_{2}}\{\left[\frac{{A_{M}}^{\;2}{\left(I_{V_{1}-M,1,case1}\right)}^{2/\varepsilon }}{\zeta^{2}{\left(D_{NLOS,1,case1}^{V_{1}-M}\right)}^{2+2/\varepsilon }}\right]\left[\frac{{D_{R}}^{\;2}{\left(I_{M-PD,1,case1}\right)}^{2/\varepsilon }}{\zeta^{2}{\left(D_{NLOS,1,case1}^{M-PD}\right)}^{2+2/\varepsilon }}\right]\\ & \times e^{-c_{E}\left(D_{NLOS,1,case1}^{V_{1}-M}+D_{NLOS,1,case1}^{M-PD}\right){\left[\frac{D_{R}}{\zeta \left(D_{NLOS,1,case1}^{V_{1}-M}+D_{NLOS,1,case1}^{M-PD}\right)}\right]}^{\varepsilon /2}}\}f_{\Delta }\left(\delta \right)d\delta , \end{array}$$

and

(A11)
$$\begin{array}{ll} I_{2} & =p_{case2}\int_{\theta_{1}}^{\theta_{2}}\{\left[\frac{{A_{M}}^{\;2}{\left(I_{V_{1}-M,1,case2}\right)}^{2/\varepsilon }}{\zeta^{2}{\left(D_{NLOS,1,case2}^{V_{1}-M}\right)}^{2+2/\varepsilon }}\right]\left[\frac{{D_{R}}^{\;2}{\left(I_{M-PD,1,case2}\right)}^{2/\varepsilon }}{\zeta^{2}{\left(D_{NLOS,1,case2}^{M-PD}\right)}^{2+2/\varepsilon }}\right]\\ & \times e^{-c_{E}\left(D_{NLOS,1,case2}^{V_{1}-M}+D_{NLOS,1,case2}^{M-PD}\right){\left[\frac{D_{R}}{\zeta \left(D_{NLOS,1,case2}^{V_{1}-M}+D_{NLOS,1,case2}^{M-PD}\right)}\right]}^{\varepsilon /2}}\}f_{\Theta }\left(\theta \right)d\theta , \end{array}$$

where $p_{case1}$ and $p_{case2}$ denotes the probability that Tx1 located to the left or right of the center axis of PD. Since the value of ε is small enough, assuming that ${\left(\frac{D_{R}}{\zeta \left(D_{NLOS,j,case}^{V_{1}-M}+D_{NLOS,j,case}^{M-PD}\right)}\right)}^{\varepsilon /2}\approx \frac{D_{R}}{\zeta \left(D_{NLOS,j,case}^{V_{1}-M}+D_{NLOS,j,case}^{M-PD}\right)}$. Then, simplify and integrate Equations (A10) and (A11) as follows:

(A12)
$$\begin{array}{ll} I_{1} & =\frac{p_{case1}\times 2{A_{M}}^{\;2}{D_{R}}^{\;2}I_{V_{1}-PD}e^{\frac{-c_{E}D_{R}}{\zeta }}}{L_{w}\zeta^{4}{\left(I_{V_{1}-M,1,case1}I_{M-PD,1,case1}\right)}^{2}}\int_{\delta_{1}}^{\delta_{2}}{\left(1-\cos2\delta \right)}^{1+2/\varepsilon }d\delta \\ & =\frac{p_{case1}\times 2{A_{M}}^{\;2}{D_{R}}^{\;2}I_{V_{1}-PD}e^{\frac{-c_{E}D_{R}}{\zeta }}}{L_{w}\zeta^{4}{\left(I_{V_{1}-M,1,case1}I_{M-PD,1,case1}\right)}^{2}}\left\{Beta\left[\frac{4{I_{V_{1}-PD}}^{\;2}}{{L_{w}}^{\;2}+4{I_{V_{1}-PD}}^{\;2}},\frac{3}{2}+\frac{2}{\varepsilon },\frac{1}{2}\right]-Beta\left[\frac{{I_{V_{1}-PD}}^{\;2}}{{L_{w}}^{\;2}+{I_{V_{1}-PD}}^{\;2}},\frac{3}{2}+\frac{2}{\varepsilon },\frac{1}{2}\right]\right\}, \end{array}$$

and

(A13)
$$\begin{array}{ll} I_{2} & =\frac{p_{case2}\times 2{A_{M}}^{\;2}{D_{R}}^{\;2}I_{V_{1}-PD}e^{\frac{-c_{E}D_{R}}{\zeta }}}{\left(L_{w}-2v_{w}\right)\zeta^{4}{\left(I_{V_{1}-M,1,case2}I_{M-PD,1,case2}\right)}^{2}}\int_{\theta_{1}}^{\theta_{2}}{\left(1-\cos2\theta \right)}^{1+2/\varepsilon }d\theta \\ & =\frac{p_{case2}\times 2{A_{M}}^{\;2}{D_{R}}^{\;2}I_{V_{1}-PD}e^{\frac{-c_{E}D_{R}}{\zeta }}}{\left(L_{w}-2v_{w}\right)\zeta^{4}{\left(I_{V_{1}-M,1,case2}I_{M-PD,1,case2}\right)}^{2}}\\ & \times \left\{Beta\left[\frac{4{I_{V_{1}-PD}}^{\;2}}{{\left(3L_{w}-2v_{w}\right)}^{2}+4{I_{V_{1}-PD}}^{\;2}},\frac{3}{2}+\frac{2}{\varepsilon },\frac{1}{2}\right]-Beta\left[\frac{{I_{V_{1}-PD}}^{\;2}}{{L_{w}}^{\;2}+{I_{V_{1}-PD}}^{\;2}},\frac{3}{2}+\frac{2}{\varepsilon },\frac{1}{2}\right]\right\}. \end{array}$$

Therefore, the average path loss of the NLOS link formed by *M* from Tx1 is as follows:

(A14)
$$\begin{array}{ll} h_{NLOS\_M,1}^{PL\_av} & =\frac{{A_{M}}^{\;2}{D_{R}}^{\;2}I_{V_{1}-PD}e^{\frac{-c_{E}D_{R}}{\zeta }}}{\left(L_{w}-v_{w}\right)\zeta^{4}}\{\frac{\left\{Beta\left[\frac{4{I_{V_{1}-PD}}^{\;2}}{{L_{w}}^{\;2}+4{I_{V_{1}-PD}}^{\;2}},\frac{3}{2}+\frac{2}{\varepsilon },\frac{1}{2}\right]-Beta\left[\frac{{I_{V_{1}-PD}}^{\;2}}{{L_{w}}^{\;2}+{I_{V_{1}-PD}}^{\;2}},\frac{3}{2}+\frac{2}{\varepsilon },\frac{1}{2}\right]\right\}}{{\left(I_{V_{1}-M,1,case1}I_{M-PD,1,case1}\right)}^{2}}\\ & +\frac{\left\{Beta\left[\frac{4{I_{V_{1}-PD}}^{\;2}}{{\left(3L_{w}-2v_{w}\right)}^{2}+4{I_{V_{1}-PD}}^{\;2}},\frac{3}{2}+\frac{2}{\varepsilon },\frac{1}{2}\right]-Beta\left[\frac{{I_{V_{1}-PD}}^{\;2}}{{L_{w}}^{\;2}+{I_{V_{1}-PD}}^{\;2}},\frac{3}{2}+\frac{2}{\varepsilon },\frac{1}{2}\right]\right\}}{{\left(I_{V_{1}-M,1,case2}I_{M-PD,1,case2}\right)}^{2}}\}. \end{array}$$

It should be noted that Tx1 and Tx2 are not symmetrical in forming the NLOS link by *M*. The average path loss of the NLOS link formed by *M* from Tx2 can be calculated as follows:

(A15)
$$\begin{array}{ll} h_{NLOS\_M,2}^{PL\_av} & =\frac{{A_{M}}^{\;2}{D_{R}}^{\;2}I_{V_{1}-PD}e^{\frac{-c_{E}D_{R}}{\zeta }}}{2^{1+2/\varepsilon }\left(L_{w}-v_{w}\right)\zeta^{4}}\{\frac{\left\{Beta\left[\frac{4{I_{V_{1}-PD}}^{\;2}}{{\left(L_{w}+2v_{w}\right)}^{2}+4{I_{V_{1}-PD}}^{\;2}},\frac{3}{2}+\frac{2}{\varepsilon },\frac{1}{2}\right]-Beta\left[\frac{{I_{V_{1}-PD}}^{\;2}}{{L_{w}}^{\;2}+{I_{V_{1}-PD}}^{\;2}},\frac{3}{2}+\frac{2}{\varepsilon },\frac{1}{2}\right]\right\}}{{\left(I_{V_{1}-M,2,case1}I_{M-PD,2,case1}\right)}^{2}}\\ & +\frac{\left\{Beta\left[\frac{{I_{V_{1}-PD}}^{\;2}}{{L_{w}}^{\;2}+{I_{V_{1}-PD}}^{\;2}},\frac{3}{2}+\frac{2}{\varepsilon },\frac{1}{2}\right]-Beta\left[\frac{{I_{V_{1}-PD}}^{\;2}}{4{L_{w}}^{\;2}+{I_{V_{1}-PD}}^{\;2}},\frac{3}{2}+\frac{2}{\varepsilon },\frac{1}{2}\right]\right\}}{{\left(I_{V_{1}-M,2,case2}I_{M-PD,2,case2}\right)}^{2}}\}. \end{array}$$

Based on (A14) and (A15), the overall average path loss closure expression for the NLOS link formed by *M* is as follows:

(A16)
$$\begin{array}{ll} h_{NLOS\_M}^{PL\_av} & =\frac{{A_{M}}^{\;2}{D_{R}}^{\;2}I_{V_{1}-PD}e^{\frac{-c_{E}D_{R}}{\zeta }}}{2\left(L_{w}-v_{w}\right)\zeta^{4}}\{\frac{\left\{Beta\left[\frac{4{I_{V_{1}-PD}}^{\;2}}{{L_{w}}^{\;2}+4{I_{V_{1}-PD}}^{\;2}},\frac{3}{2}+\frac{2}{\varepsilon },\frac{1}{2}\right]-Beta\left[\frac{{I_{V_{1}-PD}}^{\;2}}{{L_{w}}^{\;2}+{I_{V_{1}-PD}}^{\;2}},\frac{3}{2}+\frac{2}{\varepsilon },\frac{1}{2}\right]\right\}}{{\left(I_{V_{1}-M,1,case1}I_{M-PD,1,case1}\right)}^{2}}\\ & +\frac{\left\{Beta\left[\frac{4{I_{V_{1}-PD}}^{\;2}}{{\left(3L_{w}-2v_{w}\right)}^{2}+4{I_{V_{1}-PD}}^{\;2}},\frac{3}{2}+\frac{2}{\varepsilon },\frac{1}{2}\right]-Beta\left[\frac{{I_{V_{1}-PD}}^{\;2}}{{L_{w}}^{\;2}+{I_{V_{1}-PD}}^{\;2}},\frac{3}{2}+\frac{2}{\varepsilon },\frac{1}{2}\right]\right\}}{{\left(I_{V_{1}-M,1,case2}I_{M-PD,1,case2}\right)}^{2}}\\ & +\frac{\left\{Beta\left[\frac{4{I_{V_{1}-PD}}^{\;2}}{{\left(L_{w}+2v_{w}\right)}^{2}+4{I_{V_{1}-PD}}^{\;2}},\frac{3}{2}+\frac{2}{\varepsilon },\frac{1}{2}\right]-Beta\left[\frac{{I_{V_{1}-PD}}^{\;2}}{{L_{w}}^{\;2}+{I_{V_{1}-PD}}^{\;2}},\frac{3}{2}+\frac{2}{\varepsilon },\frac{1}{2}\right]\right\}}{{\left(I_{V_{2}-M,2,case1}I_{M-PD,2,case1}\right)}^{2}}\\ & +\frac{\left\{Beta\left[\frac{{I_{V_{1}-PD}}^{\;2}}{{L_{w}}^{\;2}+{I_{V_{1}-PD}}^{\;2}},\frac{3}{2}+\frac{2}{\varepsilon },\frac{1}{2}\right]-Beta\left[\frac{{I_{V_{1}-PD}}^{\;2}}{4{L_{w}}^{\;2}+{I_{V_{1}-PD}}^{\;2}},\frac{3}{2}+\frac{2}{\varepsilon },\frac{1}{2}\right]\right\}}{{\left(I_{V_{2}-M,2,case2}I_{M-PD,2,case2}\right)}^{2}}\}. \end{array}$$

## Appendix C

1. Case 1 of Tx1: Tx1 is located to the left of the center axis of the PD

As shown in Figure 5, it is known that Tx has the same height as Rx and $\delta_{i,rs}=\delta_{r,rs}$. Therefore, it can be obtained that $D_{NLOS,1,case1}^{V_{1}-RS}=D_{NLOS,1,case1}^{RS-PD}$, as well as $I_{V_{1}-RS,1}=I_{RS-PD,1}$. Thus, $D_{NLOS,1,case1}^{V_{1}-RS}$ and $D_{NLOS,1,case1}^{RS-PD}$ can be expressed as follows:

(A17)
$$D_{NLOS,1,case1}^{V_{1}-RS}=D_{NLOS,1,case1}^{RS-PD}≜X=\sqrt{{\left(\frac{L_{h_{1}}}{2}\right)}^{2}+{\left(\frac{I_{V_{1}-PD}}{2}\right)}^{2}+{L_{PD}}^{\;2}}.$$

Based on $L_{h_{1}}∼u\left(0,\frac{L_{w}}{2}\right)$ and (A17), the PDF of X can be obtained according to the probability density transformation:

(A18)
$$D_{NLOS,1,case1}^{V_{1}-RS}=X∼f_{X}(x)=\frac{8x}{L_{w}\sqrt{4x^{2}-\left({I_{V_{1}-PD}}^{\;2}+4{L_{PD}}^{\;2}\right)}},$$

where $x_{1}=\sqrt{{\left(\frac{I_{V_{1}-PD}}{2}\right)}^{2}+{L_{PD}}^{\;2}}\leq x\leq x_{2}=\sqrt{{\left(\frac{L_{w}}{4}\right)}^{2}+{\left(\frac{I_{V_{1}-PD}}{2}\right)}^{2}+{L_{PD}}^{\;2}}$.

2. Case 2 of Tx1: Tx1 is located to the right of the center axis of the PD

Similarly, in this case, $D_{NLOS,1,case2}^{V_{1}-RS}$ and $D_{NLOS,1,case2}^{RS-PD}$ can be expressed as follows:

(A19)
$$D_{NLOS,1,case2}^{V_{1}-RS}=D_{NLOS,1,case2}^{RS-PD}≜Y=\sqrt{{\left(\frac{L_{h_{1}}}{2}\right)}^{2}+{\left(\frac{I_{V_{1}-PD}}{2}\right)}^{2}+{L_{PD}}^{\;2}}.$$

Based on $L_{h_{1}}∼u\left(0,\frac{L_{w}}{2}-v_{w}\right)$ and (A19), the PDF of Y can be obtained from the probability density transformation as follows:

(A20)
$$D_{NLOS,1,case2}^{V_{1}-RS}=Y∼f_{Y}(y)=\frac{8y}{\left(L_{w}-2v_{w}\right)\sqrt{4y^{2}-\left({I_{V_{1}-PD}}^{\;2}+4{L_{PD}}^{\;2}\right)}},$$

where $y_{1}=\sqrt{{\left(\frac{I_{V_{1}-PD}}{2}\right)}^{2}+{L_{PD}}^{\;2}}\leq y\leq y_{2}=\sqrt{{\left(\frac{L_{w}-2v_{w}}{4}\right)}^{2}+{\left(\frac{I_{V_{1}-PD}}{2}\right)}^{2}+{L_{PD}}^{\;2}}$.

According to (11), (A18) and (A20), the average path loss of the NLOS link formed by *RS* from Tx1, can be expressed as follows:

(A21)
$$\begin{array}{ll} h_{NLOS\_RS,1}^{PL\_av} & =\int \left\{\sum_{case}\left\{p_{case}\left[\frac{{A_{RS}}^{\;2}{\left(I_{V_{1}-RS,1,case}\right)}^{2/\varepsilon }}{\zeta^{2}{\left(D_{NLOS,1,case}^{V_{1}-RS}\right)}^{2+2/\varepsilon }}\right]\left[\frac{{D_{R}}^{\;2}{\left(I_{RS-PD,1,case}\right)}^{2/\varepsilon }}{\zeta^{2}{\left(D_{NLOS,1,case}^{RS-PD}\right)}^{2+2/\varepsilon }}\right]e^{-c_{E}\left(D_{NLOS,1,case}^{V_{1}-RS}+D_{NLOS,1,case}^{RS-PD}\right){\left[\frac{D_{R}}{\zeta \left(D_{NLOS,1,case}^{V_{1}-RS}+D_{NLOS,1,case}^{RS-PD}\right)}\right]}^{\varepsilon /2}}\right\}\right\},\\ & \times f_{D_{NLOS,case}^{V_{1}-RS}}\left(D_{NLOS,case}^{V_{1}-RS}\right)dD_{NLOS,case}^{V_{1}-RS} \end{array}$$

where case∈{case1,case2} and $p_{case}$ denote the probability that Tx1 lies to the left or to the right of the center axis of PD. The summation in (A21) is split into two independent sub-integrals as follows:

(A22)
$$I_{1}=\int_{x_{1}}^{x_{2}}\left\{\left[\frac{{A_{RS}}^{\;2}{\left(I_{V_{1}-RS,1,case1}\right)}^{2/\varepsilon }}{\zeta^{2}{\left(D_{NLOS,1,case1}^{V_{1}-RS}\right)}^{2+2/\varepsilon }}\right]\left[\frac{{D_{R}}^{\;2}{\left(I_{RS-PD,1,case1}\right)}^{2/\varepsilon }}{\zeta^{2}{\left(D_{NLOS,1,case1}^{RS-PD}\right)}^{2+2/\varepsilon }}\right]e^{-c_{E}\left(D_{NLOS,1,case1}^{V_{1}-RS}+D_{NLOS,1,case1}^{RS-PD}\right){\left[\frac{D_{R}}{\zeta \left(D_{NLOS,1,case1}^{V_{1}-RS}+D_{NLOS,1,case1}^{RS-PD}\right)}\right]}^{\varepsilon /2}}f_{X}\left(x\right)\right\}dx,$$

and

(A23)
$$I_{2}=\int_{y_{1}}^{y_{2}}\left\{\left[\frac{{A_{RS}}^{\;2}{\left(I_{V_{1}-RS,1,case2}\right)}^{2/\varepsilon }}{\zeta^{2}{\left(D_{NLOS,1,case2}^{V_{1}-RS}\right)}^{2+2/\varepsilon }}\right]\left[\frac{{D_{R}}^{\;2}{\left(I_{RS-PD,1,case2}\right)}^{2/\varepsilon }}{\zeta^{2}{\left(D_{NLOS,1,case2}^{RS-PD}\right)}^{2+2/\varepsilon }}\right]e^{-c_{E}\left(D_{NLOS,1,case2}^{V_{1}-RS}+D_{NLOS,1,case2}^{RS-PD}\right){\left[\frac{D_{R}}{\zeta \left(D_{NLOS,1,case2}^{V_{1}-RS}+D_{NLOS,1,case2}^{RS-PD}\right)}\right]}^{\varepsilon /2}}f_{Y}\left(y\right)\right\}dy.$$

Considering the assumption ${\left(\frac{D_{R}}{\zeta \left(D_{NLOS,j,case}^{V_{1}-RS}+D_{NLOS,j,case}^{RS-PD}\right)}\right)}^{\varepsilon /2}\approx \frac{D_{R}}{\zeta \left(D_{NLOS,j,case}^{V_{1}-RS}+D_{NLOS,j,case}^{RS-PD}\right)}$, and integrating (A22) and (A23), the following can be calculated:

(A24)
$$I_{1}=\frac{16{A_{RS}}^{\;2}{D_{R}}^{\;2}{I_{V_{1}-PD}}^{\;4/\varepsilon }e^{\frac{-c_{E}D_{R}}{\zeta }}}{L_{w}\zeta^{4}P^{3/2+2/\varepsilon }}\cdot \frac{\sqrt{\pi }Gamma\left[\frac{3}{2}+\frac{2}{\varepsilon }\right]-Beta\left[\frac{4P}{4P+{L_{w}}^{\;2}},\frac{3}{2}+\frac{2}{\varepsilon },\frac{1}{2}\right]Gamma\left[2+\frac{2}{\varepsilon }\right]}{Gamma\left[2+\frac{2}{\varepsilon }\right]},$$

and

(A25)
$$I_{2}=\frac{16{A_{RS}}^{\;2}{D_{R}}^{\;2}{I_{V_{1}-PD}}^{\;4/\varepsilon }e^{\frac{-c_{E}D_{R}}{\zeta }}}{\left(L_{w}-2v_{w}\right)\zeta^{4}P^{3/2+2/\varepsilon }}\cdot \frac{\sqrt{\pi }Gamma\left[\frac{3}{2}+\frac{2}{\varepsilon }\right]-Beta\left[\frac{4P}{4P+{\left(L_{w}-2v_{w}\right)}^{2}},\frac{3}{2}+\frac{2}{\varepsilon },\frac{1}{2}\right]Gamma\left[2+\frac{2}{\varepsilon }\right]}{Gamma\left[2+\frac{2}{\varepsilon }\right]},$$

where $P={I_{V_{1}-PD}}^{\;2}+4{L_{PD}}^{\;2}$. According to (A21), as well as the integration results (A24) and (A25), the average path loss of the NLOS link formed by *RS* from Tx1, can be obtained as follows:

(A26)
$$\begin{array}{ll} h_{NLOS\_RS,1}^{PL\_av} & =\frac{8{A_{RS}}^{\;2}{D_{R}}^{\;2}{I_{V_{1}-PD}}^{\;4/\varepsilon }e^{\frac{-c_{E}D_{R}}{\zeta }}}{\zeta^{4}P^{3/2+2/\varepsilon }\left(L_{w}-v_{w}\right)Gamma\left[2+\frac{2}{\varepsilon }\right]}\{2\sqrt{\pi }Gamma\left[\frac{3}{2}+\frac{2}{\varepsilon }\right]\\ & -Gamma\left[2+\frac{2}{\varepsilon }\right]\left\{Beta\left[\frac{4P}{4P+{L_{w}}^{\;2}},\frac{3}{2}+\frac{2}{\varepsilon },\frac{1}{2}\right]+Beta\left[\frac{4P}{4P+{\left(L_{w}-2v_{w}\right)}^{2}},\frac{3}{2}+\frac{2}{\varepsilon },\frac{1}{2}\right]\right\}\}. \end{array}$$

Based on the symmetry of Tx1 and Tx2, the overall average path loss of the closed-form expression for the NLOS link formed by *RS* can be obtained as follows:

(A27)
$$\begin{array}{ll} h_{NLOS\_RS}^{PL\_av} & =\frac{8{A_{RS}}^{\;2}{D_{R}}^{\;2}{I_{V_{1}-PD}}^{\;4/\varepsilon }e^{\frac{-c_{E}D_{R}}{\zeta }}}{\zeta^{4}P^{3/2+2/\varepsilon }\left(L_{w}-v_{w}\right)Gamma\left[2+\frac{2}{\varepsilon }\right]}\{2\sqrt{\pi }Gamma\left[\frac{3}{2}+\frac{2}{\varepsilon }\right]\\ & -Gamma\left[2+\frac{2}{\varepsilon }\right]\left\{Beta\left[\frac{4P}{4P+{L_{w}}^{\;2}},\frac{3}{2}+\frac{2}{\varepsilon },\frac{1}{2}\right]+Beta\left[\frac{4P}{4P+{\left(L_{w}-2v_{w}\right)}^{2}},\frac{3}{2}+\frac{2}{\varepsilon },\frac{1}{2}\right]\right\}\}, \end{array}$$

where $P={I_{V_{1}-PD}}^{\;2}+4{L_{PD}}^{\;2}$.
